# Shaping ability of ProTaper Ultimate, ProTaper Gold, and Reciproc Blue in the mesiobuccal canals of maxillary first molars: a micro-computed tomography study

**DOI:** 10.1007/s10266-026-01381-9

**Published:** 2026-04-04

**Authors:** Özlem Sivas Yılmaz, Cangül Keskin, Ali Keleş, Amine Yiğit, İsmail Can Arı

**Affiliations:** 1 Department of Endodontics, Faculty of Dentistry, Bolu Abant İzzet Baysal University, Bolu, Turkey; 2 Department of Endodontics, Faculty of Dentistry, Ondokuz Mayıs University, Samsun, Turkey

**Keywords:** Canal transportation, Micro-computed tomography, ProTaper Gold, ProTaper Ultimate, Reciproc Blue

## Abstract

This study aimed to compare the shaping ability of ProTaper Ultimate, ProTaper Gold, and Reciproc Blue in the mesiobuccal canals of maxillary first molars using micro-computed tomography. Thirty extracted maxillary first molars with Vertucci Type IV configuration and curvatures between 10° and 20° were allocated into three groups (*n* = 10): ProTaper Ultimate, ProTaper Gold, and Reciproc Blue. All specimens underwent pre- and post-instrumentation micro-computed tomography scanning. Canal transportation, centering ratio, remaining dentin thickness, canal volume, and surface area were evaluated at predefined canal levels. Data were analyzed using one-way analysis of variance (*α* = 0.05). A significant difference was observed only at the 5 mm level of the MB1 canal, where Reciproc Blue showed a higher centering ratio than ProTaper Gold (*p* < .05). ProTaper Ultimate resulted in thinner remaining dentin in the furcation region compared with ProTaper Gold (*p* < .05), although all systems preserved more than 0.3 mm of dentin thickness. MB2 negotiation failures and instrument separation were most frequently observed in the ProTaper Gold group. Although localized differences were observed, all systems preserved canal anatomy with minimal transportation. Reciproc Blue demonstrated superior apical centering, whereas ProTaper Ultimate resulted in slightly thinner dentin in the mesial furcation area. This micro-computed tomography study provides comparative evidence on the shaping behavior of three heat-treated nickel–titanium systems in moderately curved mesiobuccal canals, and the observed differences in centering ability and dentin preservation may assist clinicians in selecting safer instrumentation strategies for complex molar anatomies.

## Introduction

The intricate anatomy of the root canal system continues to represent a major challenge in endodontic therapy. Variations in root canal morphology, particularly in multirooted teeth, predispose clinicians to procedural mishaps, such as ledge formation, canal transportation, or even perforation during instrumentation. Among these, the mesiobuccal (MB) root of maxillary first molars holds particular clinical importance because of its narrow canal diameter and abrupt curvature, especially within the coronal and middle thirds [1]. The MB root is further complicated by the frequent presence of a second canal (MB2) and inter-canal isthmuses, marked cross-sectional eccentricity, and pronounced dentin-thickness asymmetry with a distal concavity [2, 3]. These features may create zones that instruments and irrigation solutions cannot easily access, increase torsional and cyclic fatigue on files, predispose to procedural errors, such as ledging, transportation, and strip perforation, allow debris or microorganisms to persist and potentially compromise periapical healing [4]. Because these risks are driven by subtle, three-dimensional changes in canal path and dentin thickness that two-dimensional methods cannot capture reliably, an accurate volumetric technique is required to quantify shaping outcomes in MB roots. Micro-computed tomography (micro-CT) has become an established and non-destructive method for the three-dimensional, high-resolution assessment of root canal morphology and the structural alterations produced by shaping procedures, with excellent accuracy and reproducibility [5, 6]. Several micro-CT investigations have evaluated the shaping efficiency of different nickel–titanium (NiTi) systems in curved canals and reported variations in canal transportation, centering capability, and the thickness of the remaining dentin among instruments [7, 8].

Recent progress in NiTi rotary instrumentation has markedly enhanced both the efficiency and safety of root canal shaping procedures [9]. The introduction of heat-treated alloys has yielded instruments with improved flexibility and greater resistance to cyclic fatigue [10]. In the present study, ProTaper Gold (PTG), ProTaper Ultimate (PTU), and Reciproc Blue (RB) were selected as representative systems reflecting distinct thermomechanical processing techniques and kinematic concepts. PTG is produced by applying a proprietary post-machining heat treatment named Gold-wire, which increases flexibility and fatigue resistance compared with its predecessor, ProTaper Universal. Although both systems share the same geometric design, the enhanced metallurgical characteristics of PTG contribute to safer shaping performance and superior mechanical behavior [11]. The newest member of the ProTaper series PTU incorporates an optimized taper configuration, a reduced core mass, and a maximally tapered core design. These refinements enhance flexibility and mechanical performance while maintaining effective cutting efficiency [11, 12]. Conversely, the RB system functions through a single-file reciprocating motion and utilizes the Blue-wire heat treatment, which increases flexibility and fatigue resistance relative to the original Reciproc (M-Wire) system.

Several micro-CT investigations have explored the shaping behavior of different NiTi instruments in root canals exhibiting a wide range of anatomical complexities [7, 13–15]. Previous microcomputed tomography investigations have shown that RB and Reciproc exhibit comparable shaping performance under similar conditions [16]. A recent micro-CT study reported that PTU produced greater canal enlargement and dentin removal, whereas RB yielded more circular preparations and a lower percentage of unprepared walls [17].

Nevertheless, there remains a scarcity of micro-CT-based data focusing on the PTU system, particularly in the mesiobuccal canals of maxillary first molars that display moderate curvature and often include MB2 canal. The intricate anatomy of these canals makes proper instrumentation demanding and increases the likelihood of procedural deviations. Hence, additional evidence is required to better understand how instruments featuring distinct thermomechanical treatments and kinematic motions perform in canals with moderate curvature and complex morphology. To our knowledge, no study has compared PTG, PTU, and RB within a single micro-CT protocol that simultaneously quantifies canal transportation, centering ability, and remaining dentin thickness in the mesiobuccal canals of maxillary first molars. Therefore, this study evaluated and compared the shaping performance of these three contemporary NiTi systems using micro-computed tomography. The null hypothesis was that no statistically significant differences would be observed among the systems for transportation, centering, or remaining dentin thickness.

## Materials and methods

This in vitro study was approved by local Non-Interventional Clinical Research Ethics Committee (Approval No. 2024/115).

### Sample size calculation

The sample size was calculated using GPower v3.1 for Macintosh (Heinrich Heine University, Düsseldorf, Germany). An a priori power analysis was performed for the F-test family (ANOVA: fixed effects, omnibus, one-way) based on data from a previous study [18]. With an effect size (f) of 2.35, an alpha error probability of 0.05, and a statistical power (1 − β) of 0.99, a total of 10 specimens per group were determined as the required sample size to detect potential significant differences among groups.

### Sample selection

Forty-five extracted human maxillary first molars with fully developed apices, intact roots, and without caries, cracks, or previous endodontic treatment were collected from patients who had undergone extractions for periodontal, orthodontic, or prosthetic purposes. The teeth were carefully cleaned of residual soft tissue using a periodontal curette (15/16; Hu-Friedy Co., Chicago, IL, USA) and then preserved in 0.1% thymol solution at 4 °C until further use.

All specimens were initially evaluated using cone-beam computed tomography (CBCT) (I-CAT Vision™, Imaging Sciences International, Hatfield, PA, USA) to assess root canal morphology. Scanning parameters included 120 kV, 5 mA, an exposure time of 8.9 s, and a field of view measuring 16 × 13 cm with a voxel resolution of 0.3 mm. The images were processed using ImageJ software (version 1.36b; National Institutes of Health, Bethesda, MD, USA), and canal curvature was determined following the method described by Schneider [19]. In both mesiodistal (MD) and buccolingual (BL) projections, one line was drawn parallel to the canal axis in the coronal third, and another was extended from the apical foramen. The acute angle formed at their intersection represented the degree of curvature.

Following this assessment, MB roots demonstrating moderate curvature between 10° and 20° and possessing two distinct canals with a Vertucci Type IV configuration were selected [20]. To ensure sample standardization, teeth exhibiting root fractures, incomplete root formation, calcified canals, or apical foramina wider than a #10 K-file were excluded. Consequently, 30 MB roots that met all inclusion criteria were included in the final analysis.

### Sample preparation

Before commencing canal instrumentation, morphological comparability among the specimens was confirmed by analyzing the baseline morphometric data of the MB1 and MB2 canals. No statistically significant variations were observed among the groups in canal length, volume, surface area, or structure model index (SMI) (*p* > 0.05). Likewise, dentin-thickness values measured on the mesial and distal aspects at the furcation level and at 1 mm intervals up to 5 mm showed no significant differences. These results confirmed that all samples exhibited comparable morphologic characteristics and were statistically homogeneous prior to instrumentation (Table 1).

**Table 1:** Baseline morphometric parameters (mean ± SD) of the mesiobuccal canals (MB1 and MB2) showing homogeneous distribution among groups before instrumentation

	MB2			MB1		
	ProTaper Gold	ProTaper Ultimate	Reciproc Blue	ProTaper Gold	ProTaper Ultimate	Reciproc Blue
Canal length (mm)	9.45 ± 1.71^a^	9.36 ± 0.82^a^	9.41 ± 0.96^a^	9.56 ± 1.82^a^	9.10 ± 1.24^a^	9.44 ± 1.13^a^
Canal volume (mm^3^)	0.66 ± 0.38^a^	0.46 ± 0.27^a^	0.59 ± 0.20^a^	1.38 ± 0.60^a^	1.14 ± 0.06^a^	1.16 ± 0.05^a^
Surface area (mm^2^)	12.91 ± 5.55^a^	11.88 ± 4.26^a^	14.04 ± 3.62^a^	17.48 ± 5.20^a^	11.72 ± 3.39^b^	14.41 ± 5.89^ab^
SMI	2.03 ± 0.34^a^	1.95 ± 0.29^a^	1.85 ± 0.33^a^	2.21 ± 0.46^a^	2.48 ± 0.23^a^	2.26 ± 0.29^a^

Different superscript letters in the same line indicate statistically significant difference among NiTi instruments within a single canal (*p* < .05)

After homogeneity was established, the specimens were randomly divided into three experimental groups according to the NiTi system used for canal preparation: PTU, PTG, and RB (*n* = 10 MB1 and MB2 canals in each group). Randomization was performed using a computer-generated allocation sequence to ensure unbiased group assignment.

To simulate the periodontal ligament and ensure specimen stabilization during the procedures, each tooth was positioned within a custom acrylic block filled with light-body silicone impression material (Zetaplus, Zhermack SpA, Italy) to maintain standardized positioning and reproducibility throughout canal preparation and micro-CT scanning [21]. Working length (WL) was established 1 mm short of the apical foramen, determined when the tip of a #10 K-file (Dentsply Maillefer, Ballaigues, Switzerland) was visible at the apex.

All instrumentation procedures were performed by a single experienced operator following each manufacturer’s protocol. A VDW Gold Reciproc endodontic motor (VDW, Munich, Germany) was used for both continuous rotation and reciprocating motions. Throughout the shaping process, canals were irrigated with a total of 10 mL of 5.25% sodium hypochlorite (NaOCl) (Promida, manufacturer) and 5 mL of distilled water, delivered with a 30-gauge irrigation needle (Dentsply Sirona, Ballaigues, Switzerland).

### ProTaper Ultimate (PTU) group

Manual glide path preparation was performed using a #10 K-file in accordance with the standardized protocol applied across all groups. The MB1 and MB2 canals were shaped using the PTU system (Dentsply Sirona, Ballaigues, Switzerland) following the sequence of Slider, Shaper, F1, and F2 instruments. All files were used in continuous rotation at 400 rpm with a torque setting between 4 and 5.2 N·cm, as recommended by the manufacturer [22]. Instrumentation was carried out using gentle brushing motions during withdrawal to preserve the natural curvature of the canals and to promote efficient debris removal until the predetermined working length was reached.

### ProTaper Gold (PTG) group

Manual glide path preparation was performed using a #10 K-file in accordance with the standardized protocol applied across all groups. Canal preparation in this group was performed using the PTG system (Dentsply Sirona, Ballaigues, Switzerland) in the sequence of S1, S2, F1, and F2 instruments, operated in continuous rotation at 300 rpm. The torque settings were adjusted to approximately 5.1 N·cm for S1, 1.5 N·cm for S2 and F1, and 3.1 N·cm for F2, as specified by the manufacturer [23]. Each file was applied with light brushing movements along the canal walls to maintain the original curvature and minimize the risk of transportation. To prevent instrument fatigue and ensure consistency, each file was used for a single canal only.

### Reciproc Blue (RB) group

Manual glide path preparation was performed using a #10 K-file in accordance with the standardized protocol applied across all groups. The MB1 and MB2 canals were prepared using a single RB R25 file (25/0.08) (VDW, Munich, Germany) in reciprocating motion with the “Reciproc All” program of the VDW Gold Reciproc motor, as recommended by the manufacturer [24]. The file was used with a gentle in-and-out pecking motion and light apical pressure until the WL was reached.

Each NiTi instrument was strictly used for the preparation of only one canal. Separate instruments were used for MB1 and MB2 canals, even when both canals were present within the same root.

After instrumentation, all canals were rinsed with 5 mL of 17% EDTA (pH 7.4) for 1 min to remove the smear layer, followed by a final flush with 5 mL of distilled water. The canals were then dried with paper points (Dentsply Maillefer, Ballaigues, Switzerland).

### Micro-CT analyses

All samples were scanned both before and after instrumentation using a high-resolution micro-computed tomography system (SkyScan 1172, Bruker micro-CT, Kontich, Belgium). The scanning protocol was standardized at 90 kV and 105 µA with a 0.5 mm aluminum filter, a rotation step of 0.6°, and an isotropic voxel size of 16.2 µm. For each specimen, approximately 1,000 axial cross-sectional images were captured in TIFF format. Image reconstruction was carried out using NRecon software (version 1.7.1.1, Bruker micro-CT) applying a 25% beam-hardening correction, a ring artifact correction level of 10, and a smoothing factor of 3.

The pre- and post-instrumentation datasets were aligned using the three-dimensional registration module of DataViewer software (version 1.5.6.2, Bruker micro-CT). Quantitative morphometric analysis was subsequently performed with CTAn software (version 1.17.7.2, Bruker micro-CT).

### Transportation and centering ability

Within the CTAn analysis software, the canal volume (mm^3^) and surface area (mm^2^) were measured for each specimen before and after instrumentation, and the percentage increase in canal volume was subsequently calculated.

Canal transportation and centering ability were evaluated at the furcation level (FL) and at 1, 3, and 5 mm from the apical foramen. The 1-, 3-, and 5 mm levels were defined coronally from the apical foramen on the registered datasets. These parameters were determined using the mathematical formulas proposed by Gambill et al. [25]:

$${\mathrm{Transportation}} = { mid }\left( {M1 - M2} \right) \, - \, \left( {D1 - D2} \right){ mid }$$

$${\text{Centering ratio}} = \, \min \left[ {\left( {M1 - M2} \right), \, \left( {D1 - D2} \right)} \right] \, / \, \max \left[ {\left( {M1 - M2} \right), \, \left( {D1 - D2} \right)} \right]$$

M1 and D1 refer to the shortest distances between the mesial and distal canal walls and the outer surface of the root before shaping, whereas M2 and D2 denote the corresponding values after shaping.

### Dentin thickness measurement

**Fig. 1:**
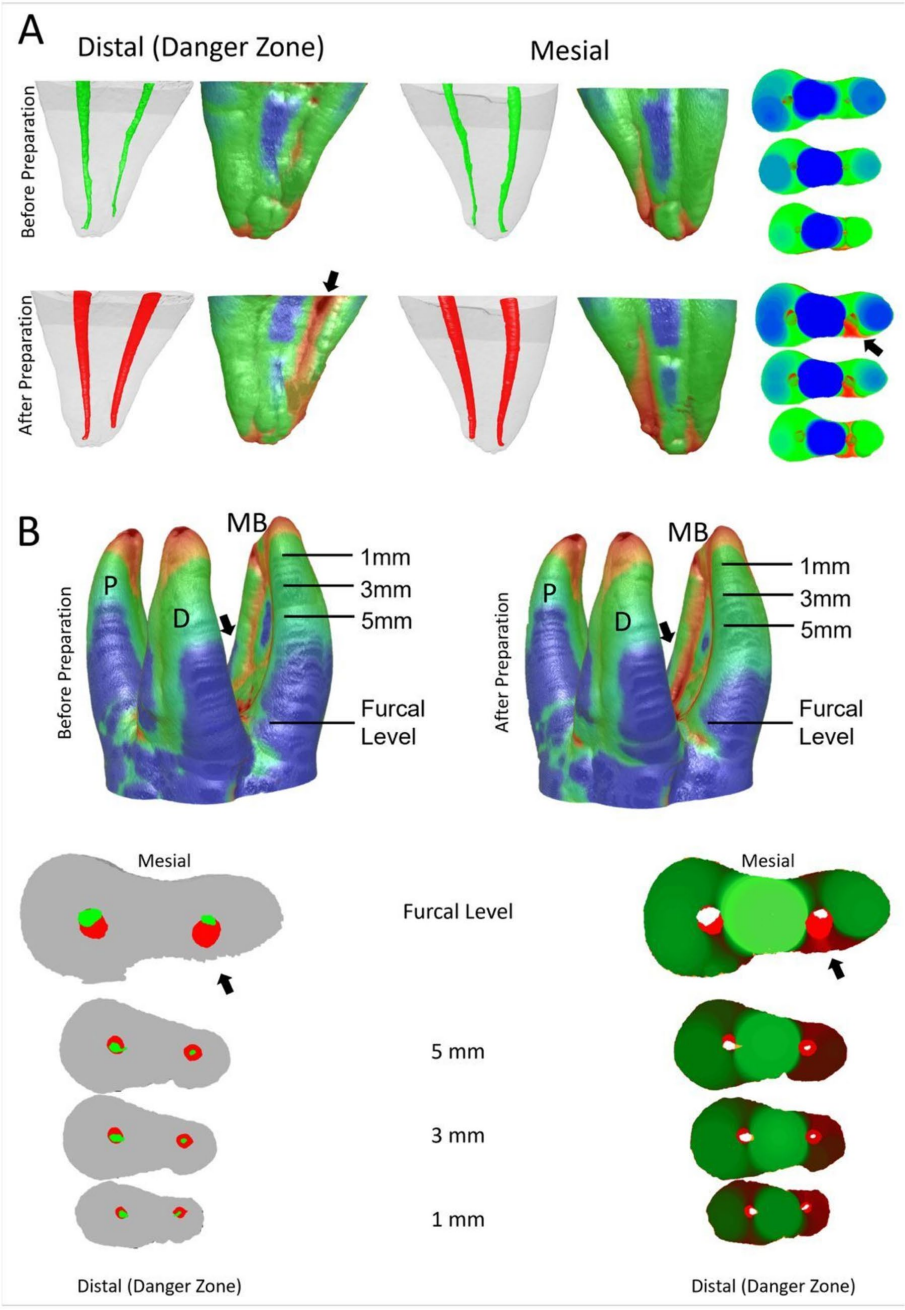
**A** Pre- and post-instrumentation three-dimensional reconstructions of the mesiobuccal root illustrating overall morphological changes. **B** Axial sections at the furcation level (FL) and at 1, 3, and 5 mm from the apical foramen. Green indicates pre-instrumentation canal boundaries, and red indicates post-instrumentation enlargement. Black arrows indicate areas of dentin reduction, particularly within the distal furcal concavity corresponding to the anatomical danger zone. Representative micro-CT reconstructions of a specimen instrumented with ProTaper Ultimate illustrating measurement methodology and general morphological patterns observed following instrumentation

Remaining dentin thickness was assessed on the mesial and distal aspects of the MB1 and MB2 canals at the furcation level (FL) and at 1, 3, and 5 mm from the apical foramen. The 1-, 3-, and 5 mm levels were defined coronally from the apical foramen on the registered datasets (Fig. 1B). Pre- and post-instrumentation micro-CT datasets were aligned using the three-dimensional registration module to ensure that measurements were taken from identical anatomical locations. At each level, the shortest distance between the canal wall and the external root surface was recorded in millimeters. The amount of dentin removed during shaping was calculated by subtracting post-instrumentation values from the corresponding baseline measurements. All assessments were performed by a single calibrated examiner to maintain measurement consistency.

### Complication rate

Procedural complications encountered during instrumentation were documented for all specimens. Two parameters were monitored: (1) failure to negotiate the MB2 canal to the established working length and (2) instrument separation. Negotiation failure was defined as the inability to advance a stainless steel #10 K-file or the designated shaping instrument to the full working length following manual glide path preparation. Instrument separation was recorded when any file fractured within the canal during use. Each complication was assigned to the corresponding file system, and the frequency of complications was compared among the groups.

### Statistical analysis

All dimensional data were recorded in millimeters and analyzed by a single calibrated examiner. To verify intra-examiner reliability, 20% of the samples were remeasured after a 2 week interval, and the reproducibility of the repeated measurements was statistically confirme**d.** All data were analyzed using IBM SPSS Statistics v26.0 (IBM Corp., Armonk, NY, USA). Normality was verified with the Shapiro–Wilk test (*p* > 0.05 for all variables); therefore, group comparisons were conducted with one-way ANOVA, followed by Tukey’s HSD for pairwise tests. Homogeneity of variances was checked with Levene’s test. Statistical significance was set at *α* = 0.05.

## Results

### Confirmation of homogenous groups

At baseline, canal length, canal volume, surface area, and structure model index (SMI) of the MB1 and MB2 canals were statistically similar among the three experimental groups (*p* > 0.05), confirming homogeneity before instrumentation (Table 1), with the exception of MB1 surface area (*p* < 0.05). Similarly, pre-instrumentation dentin thicknesses measured in the mesial and distal directions at the furcation level and at 1 mm intervals up to 5 mm showed no statistically significant differences among groups (*p* > 0.05) (Table 2).

**Table 2:** Homogeneous distribution of dentin thickness at the furcation level (FL) and at the following 5 mm levels before instrumentation

Level	Canal	Direction	ProTaper Gold	ProTaper Ultimate	Reciproc Blue
FL	MB1	Mesial	1.61 ± 0.19^a^	1.40 ± 0.08^a^	1.52 ± 0.23^a^
	MB1	Distal	1.60 ± 0.15^a^	1.58 ± 0.20^a^	1.69 ± 0.25^a^
	MB2	Mesial	1.40 ± 0.14^a^	1.36 ± 0.19^a^	1.45 ± 0.17^a^
	MB2	Distal	1.56 ± 0.32^a^	1.38 ± 0.19^a^	1.59 ± 0.10^a^
1 mm from AF	MB1	Mesial	1.51 ± 0.32^a^	1.34 ± 0.13^a^	1.41 ± 0.15^a^
	MB1	Distal	1.50 ± 0.25^a^	1.38 ± 0.23^a^	1.39 ± 0.12^a^
	MB2	Mesial	1.28 ± 0.28^a^	1.24 ± 0.20^a^	1.25 ± 0.18^a^
	MB2	Distal	1.35 ± 0.38^a^	1.08 ± 0.16^a^	1.06 ± 0.17^a^
2 mm from AF	MB1	Mesial	1.49 ± 0.31^a^	1.29 ± 0.17^a^	1.37 ± 0.14^a^
	MB1	Distal	1.44 ± 0.28^a^	1.32 ± 0.18^a^	1.32 ± 0.13^a^
	MB2	Mesial	1.25 ± 0.30^a^	1.14 ± 0.20^a^	1.11 ± 0.18^a^
	MB2	Distal	1.23 ± 0.30^a^	1.01 ± 0.13^a^	0.96 ± 0.17^a^
3 mm from AF	MB1	Mesial	1.49 ± 0.26^a^	1.29 ± 0.20^a^	1.35 ± 0.16^a^
	MB1	Distal	1.46 ± 0.42^a^	1.22 ± 0.22^a^	1.31 ± 0.15^a^
	MB2	Mesial	1.23 ± 0.34^a^	1.15 ± 0.23^a^	1.09 ± 0.21^a^
	MB2	Distal	1.20 ± 0.27^a^	1.01 ± 0.18^a^	0.96 ± 0.21^a^
4 mm from AF	MB1	Mesial	1.37 ± 0.31^a^	1.25 ± 0.24^a^	1.28 ± 0.14^a^
	MB1	Distal	1.48 ± 0.38^a^	1.26 ± 0.30^a^	1.24 ± 0.16^a^
	MB2	Mesial	1.18 ± 0.35^a^	1.06 ± 0.22^a^	1.00 ± 0.15^a^
	MB2	Distal	1.21 ± 0.25^a^	1.03 ± 0.21^a^	0.95 ± 0.21^a^
5 mm from AF	MB1	Mesial	1.27 ± 0.34^a^	1.20 ± 0.28^a^	1.26 ± 0.13^a^
	MB1	Distal	1.35 ± 0.34^a^	1.29 ± 0.31^a^	1.28 ± 0.20^a^
	MB2	Mesial	1.10 ± 0.34^a^	1.03 ± 0.28^a^	0.94 ± 0.16^a^
	MB2	Distal	0.95 ± 0.36^a^	0.95 ± 0.25^a^	0.95 ± 0.21^a^

Different superscript letters indicate statistically significant difference among instrumentation groups (*p* < .05)

### Transportation and centering ability

Following instrumentation, no statistically significant differences were found among groups in terms of total canal volume increase, surface area, or SMI in MB1 or MB2 canals (*p* > 0.05) (Table 3).

**Table 3:** Post-instrumentation morphometric changes (mean ± SD) in the MB1 and MB2 canals of maxillary first molars for each tested system

	MB1			MB2		
	ProTaper Gold	ProTaper Ultimate	Reciproc Blue	ProTaper Gold	ProTaper Ultimate	Reciproc Blue
Δ Volume (mm^3^)	1.67 ± 0.89^a^	1.74 ± 0.70^a^	1.55 ± 1.08^a^	1.28 ± 1.10^a^	1.16 ± 1.29^a^	1.88 ± 1.35^a^
Δ Surface area (mm^2^)	6.43 ± 3.87^a^	6.54 ± 3.58^a^	4.98 ± 5.60^a^	5.21 ± 4.70^a^	1.69 ± 0.66^a^	6.62 ± 6.83^a^
Δ SMI	0.42 ± 0.92^a^	0.32 ± 0.60^a^	0.31 ± 0.89^a^	0.73 ± 0.50^a^	0.93 ± 0.71^a^	0.94 ± 0.66^a^

Different superscript letters in the same line indicate statistically significant difference among instrumentation groups (*p* < .05)

For canal transportation and centering ability, the three systems demonstrated similar shaping performance at most levels (Table 4). At the 5 mm level of the MB1 canal, the RB system exhibited a significantly higher centering ratio compared with PTG, whereas no significant difference was found between RB and PTU (*p* < 0.05).

**Table 4:** Canal transportation and centering ability (mean ± SD) for MB1 canals at different measurement levels from the furcation

	Absolute transportation (mm) (transportation % toward inner curvature)			Centering ratio		
	ProTaper Gold	ProTaper Ultimate	Reciproc Blue	ProTaper Gold	ProTaper Ultimate	**Reciproc Blue**
Furcal level	0.22 ± 0.11^a^ (90)	0.15 ± 0.09^a^ (70)	0.22 ± 0.10^a^ (100)	0.65 ± 0.22^a^	0.52 ± 0.27^a^	0.39 ± 0.17^a^
1 mm from AF	0.05 ± 0.05^a^ (40)	0.06 ± 0.06^a^ (30)	0.08 ± 0.13^a^ (30)	0.41 ± 0.28^a^	0.49 ± 0.31^a^	0.60 ± 0.37^a^
3 mm from AF	0.09 ± 0.06^a^ (30)	0.03 ± 0.02^a^ (20)	0.09 ± 0.07^a^ (20)	0.42 ± 0.20^a^	0.61 ± 0.27^a^	0.53 ± 0.28^a^
5 mm from AF	0.10 ± 0.11^a^ (60)	0.08 ± 0.06^a^ (50)	0.06 ± 0.05^a^ (60)	0.33 ± 0.24^a^	0.52 ± 0.27^ab^	0.72 ± 0.18^b^

Different superscript letters indicate statistically significant difference among instrumentation groups (*p* < .05)

### Dentin thickness measurement

Post-instrumentation dentin-thickness analysis revealed a statistically significant difference at the furcation level in the mesial direction of the MB1 canal, where PTU resulted in thinner remaining dentin than PTG (*p* < 0.05) (Table 5). No significant intergroup differences were observed at other levels or in the distal direction (*p* > 0.05).

**Table 5:** Mean ± SD values of remaining dentin thickness in mesial and distal directions after preparation

		Dentin thickness in mesial direction (mm)			Dentin thickness in distal direction (mm)		
Level	Canal	ProTaper Gold	ProTaper Ultimate	Reciproc Blue	ProTaper Gold	ProTaper Ultimate	Reciproc Blue
FL	MB1	1.55 ± 0.17^a^	1.32 ± 0.12^b^	1.42 ± 0.21^ab^	1.29 ± 0.24^a^	1.35 ± 0.15^a^	1.29 ± 0.36^a^
	MB2	1.33 ± 0.18^a^	1.25 ± 0.20^a^	1.41 ± 0.15^a^	1.18 ± 0.43^a^	1.06 ± 0.37^a^	1.15 ± 0.23^a^
1 mm from AF	MB1	1.47 ± 0.26^a^	1.23 ± 0.15^a^	1.35 ± 0.13^a^	1.18 ± 0.34^a^	1.14 ± 0.19^a^	1.06 ± 0.17^a^
	MB2	1.21 ± 0.31^a^	1.11 ± 0.20^a^	1.16 ± 0.14^a^	1.03 ± 0.46^a^	0.81 ± 0.27^a^	0.72 ± 0.26^a^
2 mm from AF	MB1	1.38 ± 0.29^a^	1.18 ± 0.19^a^	1.27 ± 0.12^a^	1.23 ± 0.30^a^	1.12 ± 0.19^a^	1.09 ± 0.20^a^
	MB2	1.17 ± 0.36^a^	1.04 ± 0.23^a^	1.03 ± 0.19^a^	1.04 ± 0.48^a^	0.85 ± 0.21^a^	0.74 ± 0.22^a^
3 mm from AF	MB1	1.35 ± 0.30^a^	1.17 ± 0.19^a^	1.21 ± 0.11^a^	1.27 ± 0.35^a^	1.10 ± 0.23^a^	1.12 ± 0.23^a^
	MB2	1.11 ± 0.44^a^	1.01 ± 0.26^a^	0.98 ± 0.21^a^	1.06 ± 0.39^a^	0.89 ± 0.20^a^	0.81 ± 0.29^a^
4 mm from AF	MB1	1.18 ± 0.30^a^	1.12 ± 0.23^a^	1.14 ± 0.08^a^	1.32 ± 0.38^a^	1.16 ± 0.30^a^	1.10 ± 0.22^a^
	MB2	1.06 ± 0.43^a^	0.94 ± 0.21^a^	0.85 ± 0.21^a^	1.15 ± 0.31^a^	0.92 ± 0.20^a^	0.86 ± 0.23^a^
5 mm from AF	MB1	1.07 ± 0.29^a^	1.07 ± 0.28^a^	1.10 ± 0.12^a^	1.25 ± 0.32^a^	1.19 ± 0.32^a^	1.19 ± 0.22^a^
	MB2	1.04 ± 0.38^a^	0.94 ± 0.25^a^	0.83 ± 0.21^a^	0.91 ± 0.39^a^	0.87 ± 0.24^a^	0.90 ± 0.28^a^

Different superscript letters in the same line indicate statistically significant difference among instrumentation groups (*p* < .05)

**Fig. 2:**
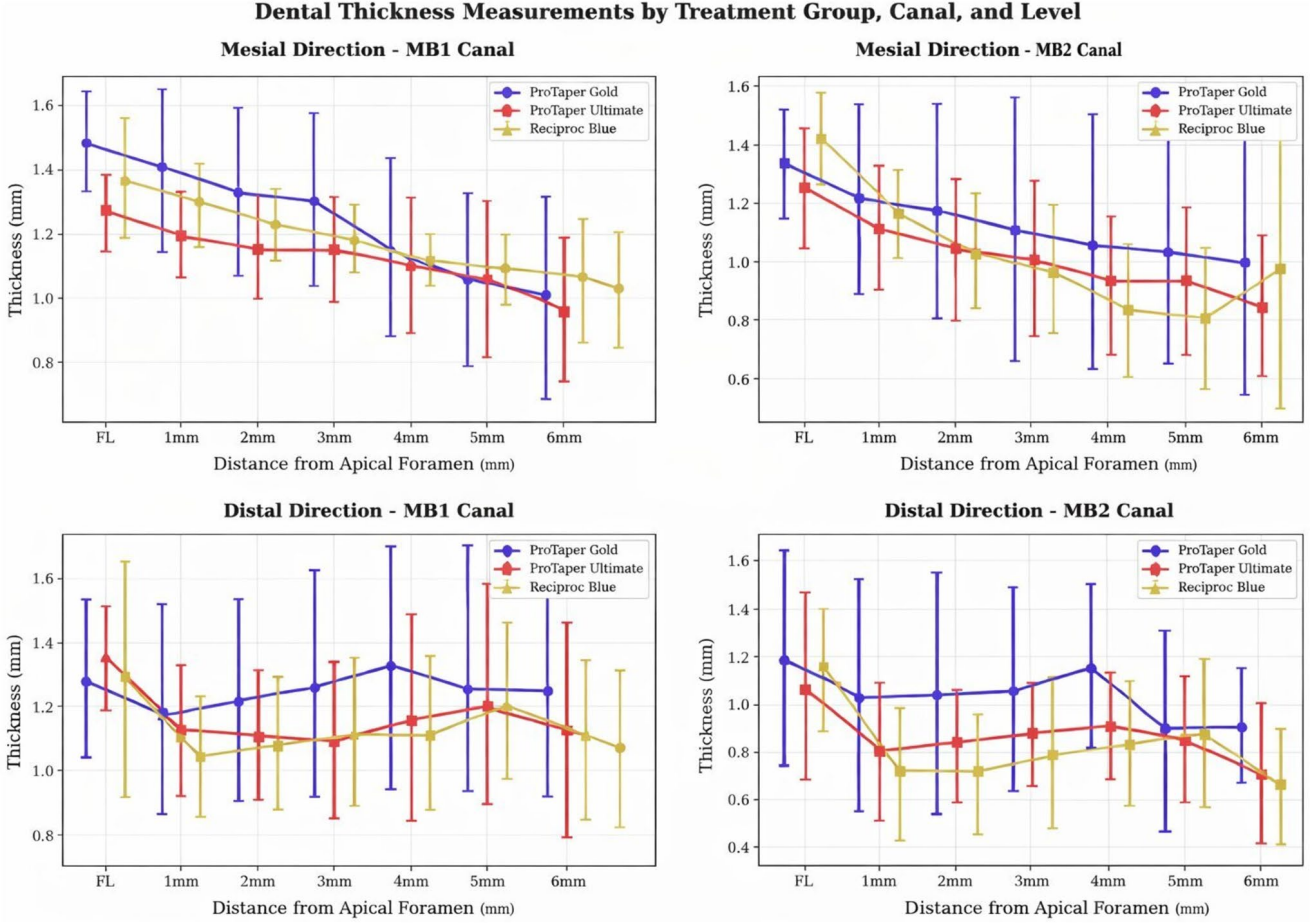
Mean dentin thickness (mm) ± SD for MB1 and MB2 canals in mesial and distal directions at each measurement level (FL, 1–6 mm from the apical foramen)

Figure 2 illustrates the dentin-thickness changes in the mesial and distal directions of the MB1 and MB2 canals at different levels from the furcation. All groups showed a gradual decrease in dentin thickness toward the apical third, with no significant intergroup differences except at the furcation level of the MB1 canal in the mesial direction, where PTU exhibited thinner remaining dentin compared with PTG (*p* < 0.05).

Representative pre- and post-instrumentation 3D reconstructions and cross-sectional images are shown in Fig. 1.

### Complication rate

In terms of clinical complications, MB2 canal negotiation failed in four cases in the PTG group, three cases in the PTU group, and only one case in the RB group. Instrument separation occurred in two canals instrumented with PTG and in one canal each prepared with PTU and RB (Table 6).

**Table 6:** Mean ± SD values of canal transportation and centering ability in MB2 canals, and distribution of procedural complications for each system

Groups	Complication (*n*)		Level	Absolute transportation (mm) (transportation % toward inner curvature)	Centering ability
ProTaper Gold	Unable to negotiate	4	FL	0.30 ± 0.21 (80)	0.20 ± 0.16
			1 mm from AF	0.01 ± 0.01 (-)	0.86 ± 0.11
	Instrument separation	2	3 mm from AF	0.03 ± 0.05 (-)	0.61 ± 0.08
			5 mm from AF	0.09 ± 0.14 (30)	0.49 ± 0.18
ProTaper Ultimate	Unable to negotiate	3	FL	0.20 ± 0.20 (70)	0.43 ± 0.29
			1 mm from AF	0.04 ± 0.05 (40)	0.46 ± 0.28
	Instrument separation	1	3 mm from AF	0.04 ± 0.06 (20)	0.64 ± 0.21
			5 mm from AF	0.06 ± 0.06 (60)	0.72 ± 0.15
Reciproc Blue	Unable to negotiate	1	FL	0.43 ± 0.22 (80)	0.16 ± 0.17
			1 mm from AF	0.04 ± 0.07 (10)	0.68 ± 0.26
	Instrument separation	1	3 mm from AF	0.04 ± 0.06 (20)	0.49 ± 0.13
			5 mm from AF	0.06 ± 0.06 (60)	0.49 ± 0.28

## Discussion

This micro-CT investigation aimed to evaluate shaping behavior in moderately curved mesiobuccal canals by comparing instruments with different kinematic strategies, geometric designs, and metallurgical characteristics. At the pre-instrumentation stage, morphometric variables, such as canal length, volume, surface area, structure model index (SMI), and dentin thickness, did not differ significantly among the groups. This indicated that all specimens were morphologically comparable before shaping. Such baseline uniformity helped to minimize anatomical variability, ensuring that any observed post-instrumentation outcomes could be linked to the design and metallurgical features of the tested instruments rather than differences in root morphology [26, 27]. Establishing this type of standardization is particularly important in micro-CT analyses, because even slight preoperative variations in curvature or dentin thickness can influence shaping patterns and transportation behavior. In the current study, groups were generally similar for canal transportation, centering ratio, or residual dentin thickness; however, two localized differences emerged as RB achieved a higher centering ratio than PTG at MB1–5 mm, and PTU left thinner dentin than PTG at the furcation-related mesial section of MB1. Thus, the null was not uniformly supported.

Overall, most evaluated outcomes demonstrated comparable shaping behavior among the tested instruments, with only localized statistically significant differences observed. Accordingly, the present findings should be interpreted as incremental and largely confirmatory, supporting previous micro-CT evidence indicating that contemporary heat-treated NiTi systems tend to exhibit similar shaping performance in moderately curved canals. The contribution of this study is, therefore, best positioned as a comparative confirmation within a controlled anatomical model, rather than as evidence of transformative differences among instrument systems.

Although no overall significant differences were observed among the systems, at the 5-mm level of the MB1 canal, the RB system exhibited a significantly higher centering ratio than PTG, while no statistical difference was detected between RB and PTU. The higher centering ratio observed with the reciprocating instrument at the 5 mm level may be associated with the mechanical characteristics of reciprocating kinematics. As a result, torsional stress accumulation is reduced and the canal path remains more centered [28, 29]. In addition, the proprietary Blue heat treatment confers greater flexibility and resistance to cyclic fatigue, enabling the file to adapt more closely to the canal curvature and to maintain centering even in moderately curved roots [28, 30]. All of the NiTi systems tested were able to preserve the original canal curvature, exhibiting minimal transportation and a comparable ability to remain centered. This finding suggests that, within the limitations of moderately curved MB canals, contemporary heat-treated NiTi instruments may exhibit comparable shaping behavior irrespective of kinematic motion. Similar trends have been reported in recent micro-CT investigations, which showed that current heat-treated NiTi instruments provide equivalent shaping efficiency regardless of whether they operate in continuous rotation or reciprocation [31, 32]. Metallurgical advancements in thermomechanically processed alloys may also have contributed to this outcome. Improved flexibility and controlled memory properties enable better adaptation of instruments to curved canal walls. [33]. Because taper configuration was standardized, the observed variations are more plausibly attributed to differences in flexibility, stress distribution patterns, and cutting mechanics associated with instrument design and alloy treatment.

According to Alovisi et al. (2025), PTU preserved more dentin and showed improved centering compared with PTG. In our study, a localized, surface-specific effect was observed at the furcation-related mesial section of MB1, where PTU left thinner dentin than PTG, indicating slightly less conservative shaping at that surface. Elsewhere, remaining dentin thickness did not differ among systems [34]. These differences, where present, are plausibly related to PTU’s heat-treated metallurgy and parallelogram cross-section, rather than taper variation [11]. On the other hand, the finding that RB exhibited the highest centering ratio at the 5-mm level of the MB1 canal indicates a superior ability of this system to maintain the original canal path in the apical region. This advantage is directly attributable to the mechanical characteristics of reciprocating kinematics; by reducing continuous rotational stress and minimizing the screw-in effect, reciprocation promotes more centered preparation patterns, particularly in curved canals [28, 29, 33].

At the furcation level, on the mesial aspect of the MB1 canal, the PTU system produced a thinner layer of remaining dentin compared with PTG. Although this difference was detected only at a single cross-section, the localized thinning can be anatomically explained. Importantly, the mesial aspect of the mesiobuccal root typically presents a greater bulk of dentin compared to the distal furcal concavity, which constitutes the actual danger zone [3]. This outcome may also be associated with the cutting design of the Ultimate finishing files. This localized thinning may be related to differences in cutting efficiency and cross-sectional geometry rather than a generalized effect of the instrument system. [11]. Comparable tendencies have been described in other micro-CT investigations, where newer rotary systems featuring refined metallurgy and enhanced cutting geometry were found to remove greater amounts of dentin in curved roots, especially near the furcation area where stress concentration is higher [28, 34]. Despite this localized variation, all groups maintained a minimum residual dentin thickness greater than 0.3 mm, indicating that none of the systems jeopardized root strength or increased the likelihood of strip perforation. Importantly, although statistically significant differences were detected at isolated measurement levels, the magnitude of these variations was limited. Therefore, while statistically significant, these localized dimensional changes are clinically negligible and do not compromise the biomechanical stability of the root.

From a clinical perspective, negotiation of the MB2 canal was less successful with continuously rotating instruments compared with the reciprocating system. This finding may be interpreted in relation to the anatomical challenges associated with MB2 anatomy. The narrow canal diameter, abrupt curvature, and restricted straight-line access increase the likelihood of instrument engagement and canal deviation. Reciprocating kinematics may offer a mechanical advantage under such conditions. By reducing continuous rotational contact between the instrument and canal walls, this motion may limit screw-in forces and decrease the risk of taper lock. This motion pattern has been associated with more centered preparation behavior and reduced canal deviation in curved canals, as demonstrated in the previous micro-CT investigations [28, 33, 35]. In addition, enhanced alloy flexibility may further contribute to negotiation behavior. Greater flexibility allows improved conformation to canal curvature while reducing restoring forces that tend to straighten the instrument within confined anatomical pathways. Differences in canal negotiation and complication rates may, therefore, reflect the mechanical characteristics of the kinematic motion and the metallurgical properties of the instruments rather than a system-specific effect.

Instrument separation was observed more frequently in the continuously rotating instrument group, whereas both the reciprocating and heat-treated rotary systems exhibited only isolated events. Given the low overall incidence of separation, these findings should be interpreted cautiously; however, the observed pattern is consistent with the previous mechanical investigations reporting reduced torsional stress accumulation under reciprocating motion [36, 37].

Some limitations of this study should also be acknowledged. Because the investigation was conducted on extracted teeth, the results may not exactly reproduce the biological and mechanical conditions found in vivo. In addition, all shaping procedures were carried out by one experienced operator. Although this eliminated inter-operator variability, it might restrict the generalizability of the outcomes. The study design was limited to moderately curved mesiobuccal roots with Vertucci Type IV configuration; thus, the conclusions may not extend to canals with greater curvature or different anatomical patterns. Moreover, the analysis was confined to geometric variables and did not include clinical aspects, such as debris extrusion, cleaning effectiveness, or dentinal microcrack formation. Future investigations should aim to confirm these findings under in vivo or simulated clinical conditions. The use of finite-element modeling, photoelastic analysis, real-time torque and force recording, and fatigue resistance testing could further clarify mechanical behavior and stress distribution during shaping. Therefore, the present conclusions primarily apply to moderately curved mesiobuccal canals with a Vertucci Type IV configuration and may not be directly extrapolated to canals with more severe curvature or different anatomical patterns.

## Conclusions

Within the limitations of this micro-CT analysis, the findings indicate that PTU, PTG, and RB exhibit comparable and predictable shaping performance in moderately curved mesiobuccal root canals of maxillary first molars. The superior centering ability observed for RB at the apical third and the slightly thinner dentin remaining with PTU in the mesial furcation area represent localized differences rather than broad performance trends. All systems preserved dentin thickness above clinically acceptable thresholds and demonstrated low rates of procedural complications. RB also achieved the highest success in MB2 canal negotiation. Overall, the three systems provided safe and clinically acceptable shaping outcomes in mesiobuccal canals.

## Data Availability

The data that support the findings of this study are available from the corresponding author upon reasonable request.
